# The incremental value of *Mycobacterium tuberculosis* trace nucleic acid detection in CT-guided percutaneous biopsy needle rinse solutions for the diagnosis of tuberculosis

**DOI:** 10.3389/fmicb.2024.1335526

**Published:** 2024-02-08

**Authors:** Zihui Li, Bing Wang, Boping Du, Qi Sun, Dongpo Wang, Rongrong Wei, Chenghai Li, Chuanzhi Zhu, Hongyan Jia, Aiying Xing, Zongde Zhang, Liping Pan, Dailun Hou

**Affiliations:** ^1^Laboratory of Molecular Biology, Beijing Key Laboratory for Drug Resistant Tuberculosis Research, Beijing Chest Hospital, Capital Medical University, Beijing Tuberculosis and Thoracic Tumor Research Institute, Beijing, China; ^2^Department of Radiology, Beijing Chest Hospital, Capital Medical University, Beijing Tuberculosis and Thoracic Tumor Research Institute, Beijing, China

**Keywords:** tuberculosis, biopsy, biopsy needle rinse solution, digital PCR, diagnosis, computed tomography (CT)

## Abstract

**Introduction:**

Tuberculosis (TB) diagnosis still faces challenges with high proportion of bacteriologic test negative incidences worldwide. We assessed the diagnostic value of digital PCR (dPCR) analysis of ultramicro *Mycobacterium tuberculosis* (*M.tb*) nucleic acid in CT-guided percutaneous biopsy needle rinse solution (BNRS) for TB.

**Methods:**

BNRS specimens were consecutively collected and total DNA was purified. The concentrations of *M.tb*-specific IS*6110* and IS*1081* were quantified using droplet dPCR. The diagnostic performances of BNRS-dPCR and its sensitivity in comparison with conventional tests were analyzed.

**Results:**

A total of 106 patients were enrolled, 63 of whom were TB (48 definite and 15 clinically suspected TB) and 43 were non-TB. The sensitivity of BNRS IS*6110* OR IS*1081*-dPCR for total, confirmed and clinically suspected TB was 66.7%, 68.8% and 60.0%, respectively, with a specificity of 97.7%. Its sensitivity was higher than that of conventional etiological tests, including smear microscopy, mycobacterial culture and Xpert using sputum and BALF samples. The positive detection rate in TB patients increased from 39.3% for biopsy AFB test alone to 73.2% when combined with BNRS-dPCR, and from 71.4% for biopsy *M.tb* molecular detection alone to 85.7% when combined with BNRS-dPCR.

**Conclusion:**

Our results preliminarily indicated that BNRS IS*6110* OR IS*1081*-dPCR is a feasible etiological test, which has the potential to be used as a supplementary method to augment the diagnostic yield of biopsy and improve TB diagnosis.

## 1 Introduction

Tuberculosis (TB) was the predominant cause of mortality attributable to a single infectious agent prior to the coronavirus (COVID-19) pandemic. Globally in 2021, an estimated 10.6 million people fell ill with TB, and 1.6 million people died from the disease (World Health Organization, 2022).

An essential step in the pathway of global TB infection control is rapid and accurate testing to diagnose TB. Currently, laboratory diagnosis of *Mycobacterium tuberculosis* (*M.tb*) infection primarily relies on the detection of *M.tb* and *M.tb*-specific immune responses. The latter, such as tuberculin skin test (TST) and interferon-γ release assay (IGRA), serves as an auxiliary diagnostic tool for TB but possess limitations when applied to immunocompromised individuals (Redelman-Sidi and Sepkowitz, 2013; Nguyen et al., 2018). Testing for *M.tb* or its nucleic acids or antigens is crucial as it allows people to get an etiological diagnosis and start on effective treatment regimen as early as possible. The traditional smear microscopy with Ziehl-Nielsen staining is widely used with low sensitivity. Mycobacterial culture is more sensitive, but its application is greatly limited by the longtime of return results (10 days - 8 weeks) and biosafety requirements. In recent years, the development and application of nucleic acid amplification-based tests (NAATs) of *M.tb*, such as conventional polymerase chain reaction (PCR), quantitative real-time PCR (qPCR), loop-mediated isothermal amplification (LAMP), as well as Xpert MTB/RIF (Xpert) and Xpert Ultra recommended by WHO, have improved the microbiological diagnosis of TB (Lawn et al., 2013; Detjen et al., 2015; Dorman et al., 2018; Sabi et al., 2018; Gong et al., 2023). However, despite the availability of these methods, the worldwide percentage of bacteriological confirmation of pulmonary TB remains at only 63% in 2021 due to complex factors such as sensitivity, testing cost and experimental facilities (World Health Organization, 2022). Therefore, more etiological methods are urgently needed to enhance TB diagnosis.

Biopsy plays a crucial role in establishing a definitive diagnosis for patients who have undergone all routine procedures but remain undiagnosed (Xu et al., 2023; Zhao et al., 2023). Usually, the minimal cellular residues adhering to the puncture needle are discarded along with the used needle following the biopsy operation. Considering the trajectory of the puncture needle, the outer surface of the needle contacts a larger area of lesion tissue compared to the needle groove housing the biopsy tissue. Consequently, residual cells on needle's outer wall may harbor pathogenic evidence that differs from the biopsy tissue. Previous studies have demonstrated the role of this kind of biopsy needle rinse solutions (BNRS) in enhancing lung cancer diagnosis and multiple genetic analyses (Sakairi et al., 2014; Lan et al., 2021). Whether this type of ultra-microsample can be used to provide the etiological evidence of TB has not been reported.

Digital PCR (dPCR) is a robust technique employed for the absolute quantification of trace amounts of nucleic acids. Compared to qPCR, dPCR offers advantages such as quantification without the need for a standard curve, improved precision, and enhanced tolerance to PCR inhibitors (Kuypers and Jerome, 2017). It has demonstrated good efficacy in improving the diagnosis of paucibacillary TB, including tuberculous meningitis and pleurisy (Li et al., 2020, 2022). The aim of this study was to assess the value of computed tomography (CT) - guided percutaneous BNRS-dPCR analysis in the diagnosis of TB.

## 2 Materials and methods

### 2.1 Study participants

We conducted the cross-sectional study from 2021 to 2022 at Beijing Chest Hospital, Beijing, China. Patients who met the inclusion and exclusion criteria were consecutively enrolled. The inclusion criteria were as follows: (1) age ≥ 18 years; (2) imaging showed isolated or multiple lesions suggesting TB; (3) received doctor's advice for CT-guided percutaneous biopsy to confirm diagnosis; (4) provided informed consent for the study. The exclusion criteria were as follows: (1) uncorrectable coagulation abnormalities; (2) severe pulmonary arterial hypertension; (3) anatomically or functionally isolated lung; (4) pulmonary bulla, chronic obstructive pulmonary disease, emphysema, and pulmonary fibrosis; (5) mechanical ventilation; (6) imaging findings of pulmonary hydatidosis, which may increase the risk of allergy; (7) uncontrolled cough or other conditions that may affect biopsy. The sample size was calculated using PASS 11 software, with area under curve (AUC) 0 (H0) = 0.5, AUC1 (H1) = 0.7 (set according to preliminary experimental results), α = 0.025, β = 0.1, sample allocation ratio = 1.0 and one-sided test. At least 41 cases needed to be included in each group. Some of the following tests were performed to help make a final diagnosis: formalin-fixed paraffin-embedded (FFPE) biopsy tissue tests including routine pathology, acid-fast bacilli (AFB) detection and molecular pathology (detection of *M.tb* nucleic acids) (Shengxiang, Changsha, Hunan, China); *M.tb* tests including smear microscopy for AFB, mycobacterial culture and Xpert (Cepheid, Sunnyvale, CA, USA) using sputum, bronchoalveolar lavage fluid (BALF) or pus samples; blood tests related to *M.tb* infection including IGRA (X.DOT-TB; Signature Biotechnology, Foshan, Guangdong, China) and *M.tb* antibody detection (Huian, Shenzhen, Guangdong, China). All of the commercial assays were performed according to the manufacturer's instructions. This study was approved by the Ethics Committee of Beijing Chest Hospital, Capital Medical University (ethical approval number: 2021 clinical trial review-scientific research-no. 37). Written informed consent was acquired from each participant before enrollment or any study procedure.

### 2.2 Categorization of patients

Patients were divided into two groups: (1) TB group: composite reference standard (CRS) was used as gold standard according to the diagnostic criteria (WS288-2017) (National Health and Family Planning Commission of the People's Republic of China, 2018), which was composed of clinical, laboratory, histopathological and radiological features. Confirmed TB: imaging examination was positive, at least one etiological test was positive, and nontuberculous mycobacterial disease was excluded by species identification or *M.tb*-specific molecular detection. Clinically suspected TB: with positive imaging findings and positive immunological results or typical clinical manifestations. Confirmed TB by molecular pathology: confirmed TB with positive *M.tb* molecular detection using FFPE biopsy tissue. Etiological tests included AFB detection, mycobacterial culture or commercial NAAT for *M.tb* using sputum, BALF, pus or biopsy samples. Immunological tests included blood IGRA or *M.tb* antibody detection. (2) Non-TB group: an alternative diagnosis was made, without convincing signs of *M.tb* infection.

### 2.3 CT-guided percutaneous biopsy

Percutaneous biopsy was performed by radiologists under the guidance of a 64-detector row CT scanner (Light Speed, VCT, GE, Milwaukee, WI, USA). Depending on the location of the lesion, the patient was placed in the supine or prone position and instructed to remain motionless throughout the entire puncture process. After the patient was in a stable state, the needle entry point was selected under the guidance of CT. Following skin disinfection, the patient was locally anesthetized with 2% lidocaine. Then a small incision at the injection point was made using a disposable core biopsy instrument (MC1810, MC1816, BARD, Murray Hill, NJ, USA) to retrieve specimens. After the biopsy procedure, all visible tissue specimens were removed and placed in formalin for routine histopathological examination. The front part of the used needle was rinsed in a 10-mL aseptic phosphate buffered saline (PBS) tube several times without touching the tube wall. This tube containing BNRS was then sent to the laboratory for further testing.

### 2.4 BNRS sample processing and DNA extraction

The tubes containing BNRS were centrifuged at 4000 rpm for 10 min at room temperature, and the sediments and 800 μL supernatant were frozen at −80 °C. They were mixed and transferred into a 2 ml Lysing Matrix B tubes (116911050, MP, California, USA) and added 200 μL Buffer ATL (939011, Qiagen, Hilden, Germany) with 0.67% Reagent DX (19088, Qiagen, Hilden, Germany). The tubes were vortexed on the FastPrep-24 instrument (116004500, MP, California, USA) applying a velocity of 6.5 m/s for three times 45 s with a 5 min intermission. After centrifugation, 400 μL supernatants were transferred into fresh tubes for DNA extraction using DNeasy Blood and Tissue Kits (69506, Qiagen, Hilden, Germany) with an elution volume of 50 μL. DNA samples were extracted in batches and stored at −80 °C until dPCR detection.

### 2.5 Digital PCR analysis

Conserved DNA sequences in *M.tb* complex, insertion sequence IS*6110* and IS*1081*, were used as detection targets in this study. The primers for amplification, oligonucleotide probes and dPCR assay procedures have been previously optimized and described in Li et al. (2022). The 20-μL reaction mixtures (10 μL ddPCR^TM^ supermix for probes, 0.9 μM each primer, 0.2 μM each probe, 0.3 U uracil-N-glycosylase, DNA samples without dilution) and 70-μL droplet generation oil were added in cartridges and droplets were generated using QX200 Droplet Generator (Bio-Rad). After droplet emulsions were transferred into the 96-well plate and PCR procedure, the fluorescence signals of each droplet in each well was acquired using QX200 Droplet Reader (BioRad) with FAM and HEX/VIC channels. Data analysis was performed using QuantaSoft Version 1.7.4.0917 (BioRad) and the absolute quantity of target DNA in each well were automatically calculated based on the Poisson distribution. No-template negative control and *M.tb* H37Rv DNA positive control were adopted in each assay, which was used to set the threshold manually based on their fluorescence amplitudes. The number of copies per 20-μL reaction mixture was calculated as the average of two independent dPCR results, with each result in duplicate.

### 2.6 Statistical analysis

All statistical analysis was performed using the software SPSS version 25.0 (IBM, Armonk, NY, USA). Continuous variables were compared using Mann-Whitney *U-*test or Wilcoxon test, as appropriate. Categorical variables were tested by Chi-square test. Receiver operating characteristic (ROC) curves were constructed by plotting the rate of sensitivity against the rate of (1-specificity) over a range of cut-off values of dPCR. Diagnostic performance was expressed in terms of sensitivity, specificity, positive likelihood ratio (LR+), negative likelihood ratio (LR-), positive predictive value (PPV), negative predictive value (NPV) and AUC. All tests were two-sided and *P* < 0.05 were considered statistically significant.

## 3 Results

### 3.1 Participant characteristics

**Figure 1:**
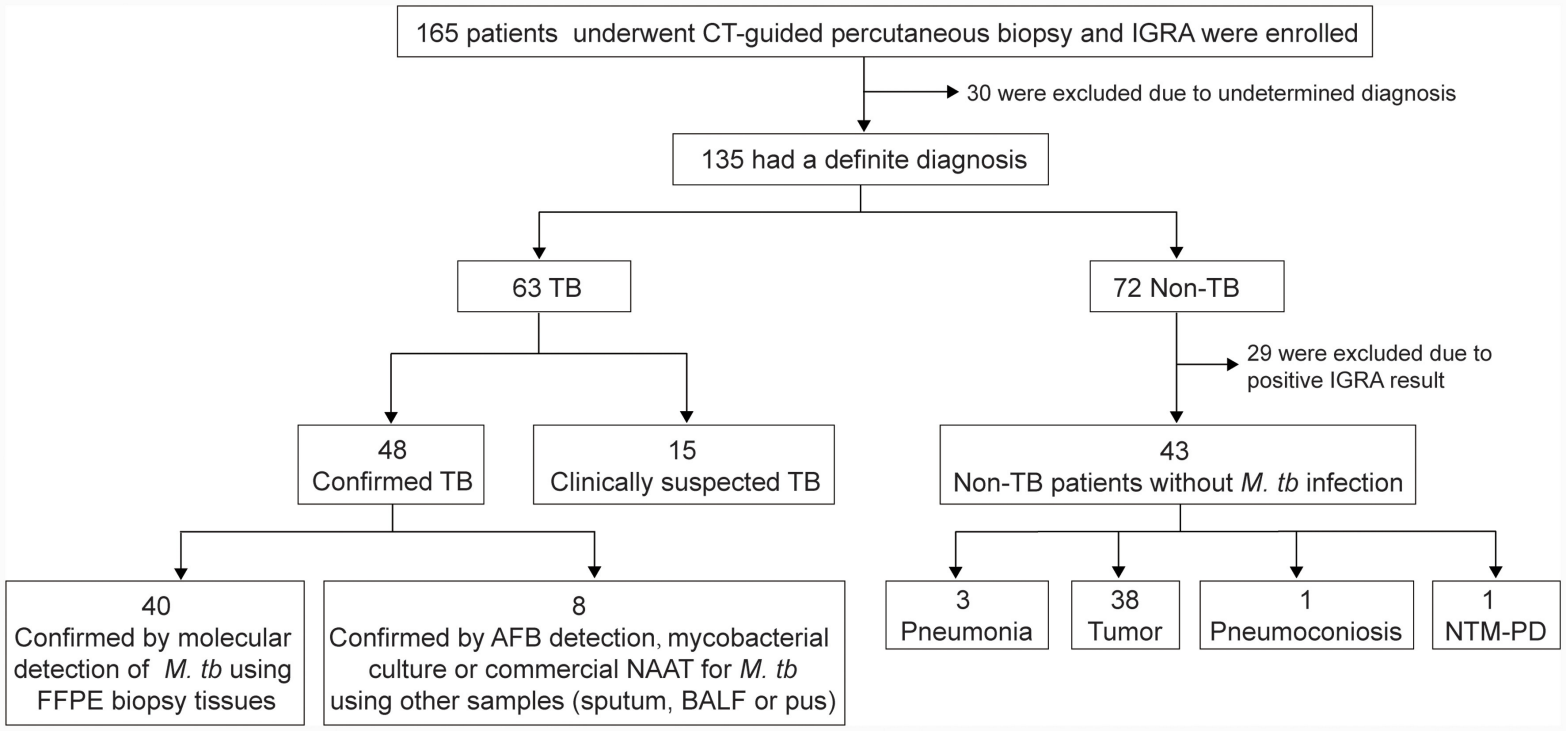
Recruitment of the participants. IGRA, interferon-gamma release assay; FFPE, formalin-fixed paraffin-embedded; BALF, bronchoalveolar lavage fluid; AFB, acid-fast bacilli; NAAT, nucleic acid amplification test; NTM-PD, nontuberculous mycobacterial pulmonary disease.

A total of 165 subjects who underwent CT-guided percutaneous biopsy were prospectively enrolled and 30 patients were excluded due to undetermined diagnosis. Among the 135 patients, 63 were diagnosed as TB and 72 as non-TB. Twenty-nine patients were excluded from non-TB group due to positive IGRA result indicating concurrent infection with *M.tb*. Therefore, the final sample size for analysis comprised 106 patients, including 63 TB patients (48 definite TB cases and 15 clinically suspected TB cases) and 43 non-TB patients without *M.tb* infection. Among the 43 non-TB patients, there were 38 cases of tumor, 3 cases of pneumonia, 1 case of pneumoconiosis, and 1 case of non-tuberculous mycobacterial pulmonary disease (Figure 1). Overall, the study population consisted of 65 male patients (61%), with a median age of 53.5 years (range: 18 to 83). TB patients were younger than non-TB patients (*P* < 0.0001). The biopsy tissues primarily originated from the lungs, as well as the pleura, peritoneum, chest wall, and mediastinum. Demographic and basic clinical characteristics of the study population are presented in Table 1.

**Table 1:** Demographic and clinical characteristics of the participants.

**Characteristics**	**Total patients (*N =* 106)**	**TB (*N =* 63)**	**Non-TB (*N =* 43)**	***P*-value**
Age - median (range), yr	53.5 (18, 83)	44 (18, 80)	64 (32, 83)	< 0.0001
Male sex - no. (%)	65 (61)	41 (65)	24 (56)	0.336
BMI ^a^ - mean ± SD	22.8 ± 3.8	22.3 ± 3.3	23.5 ± 4.2	0.097
Biopsy site				0.082 ^b^
Lung	88	49	39	
Pleura	11	10	1	
Peritoneum	2	2	0	
Chest wall	2	2	0	
Mediastinum	3	0	3	

^a^BMI, body-mass index, is the weight in kilograms divided by the square of the height in meters. ^b^Comparison of the proportion of biopsy sites in lung and non-lung between TB and non-TB group (Pearson's Chi-square test).

### 3.2 Results of dPCR in detection of *M.tb* nucleic acids in BNRS

**Figure 2:**
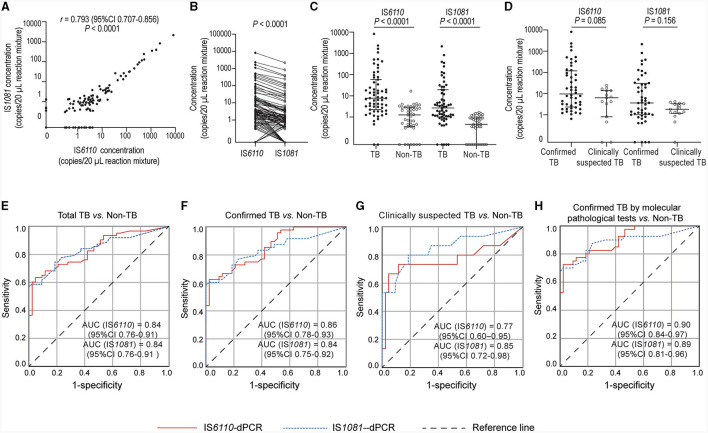
Quantification of *M.tb* nucleic acids in biopsy needle rinse solutions (BNRS) samples by dPCR and receiver operating characteristic (ROC) curves analysis. **(A, B)** show the correlation (Spearman correlation test) and the differences (Wilcoxon test) in the number of copies detected between IS*6110*- and IS*1081*- dPCR, respectively. **(C)** shows IS*6110* and IS*1081* copies detected in TB and non-TB group, respectively (Mann–Whitney *U*-test). **(D)** shows IS*6110* and IS*1081* copies detected in confirmed and clinically suspected TB patients, respectively (Mann–Whitney *U*-test). All copy numbers are obtained from the average of two independent dPCR results, with each result in duplicate. Results are considered significant when *P* < 0.05. **(E–H)** ROC curve analysis of IS*6110*- and IS*1081*- dPCR for the diagnosis of total TB, confirmed TB, clinically suspected TB and confirmed TB by molecular pathological tests, respectively. AUC, area under ROC curve; CI, confidence interval.

The IS*6110-* and IS*1081*- targeted tests exhibited a strong positive correlation (*r* = 0.793, *P* < 0.0001, Figure 2A). In most cases, the number of IS*6110* detected was higher than that of IS*1081* (*P* < 0.0001, Figure 2B). The number of copies detected in TB group was significantly higher than that in non-TB group: median (25% percentile, 75% percentile), IS*6110*, 7.8 (2.2, 58.0) vs. 1.3 (0.4, 2.9) copies/20 μL reaction mixture, *P* < 0.0001; IS*1081*, 2.7 (1.0, 19.1) vs. 0.5 (0.0, 0.9) copies/20 μL reaction mixture, *P* < 0.0001 (Figure 2C). The number of copies detected in confirmed TB group was slightly higher than that in clinically suspected TB group; however, the difference was not significant due to a relatively small number of cases (Figure 2D).

### 3.3 Performance of BNRS-dPCR in the diagnosis of TB

The ability to detect *M.tb* DNA in BNRS was assessed using ROC analysis. The overall area under the ROC curve (AUC) of IS*6110-*dPCR was nearly identical to that of IS*1081-*dPCR [both 0.84, 95% confidence interval (CI): 0.76-0.91, *P* = 0.93, Figure 2E]. There was no significant difference in the AUC of dPCR between patients with confirmed TB and those with clinically suspected TB (Figures 2F, G). The AUC of dPCR in patients with confirmed TB by molecular pathology was the highest (IS*6110*, 0.90, 95% CI 0.84-0.97; IS*1081*, 0.89, 95% CI 0.81-0.96, Figure 2H). The diagnostic performance of BNRS-dPCR assay for TB is presented in Table 2. In this study, the optimal cut-off values to ensure high specificity and then the maximum sum of sensitivity and specificity were defined as 4.2 (IS*6110*) and 1.7 (IS*1081*) copies/20 μL reaction mixture, respectively. An IS*6110* OR IS*1081-*dPCR result was considered “positive” if either IS*6110* or IS*1081* > their cut-off values, and ‘negative' if both IS*6110* and IS*1081* ≤ their cut-off values. Its sensitivity was higher than that of single target-dPCR and IS*6110 and* IS*1081-*dPCR, while maintaining a relatively high specificity. The sensitivity of IS*6110* OR IS*1081-*dPCR for total, confirmed and clinically suspected TB was 66.7%, 68.8% and 60.0%, respectively, with a specificity of 97.7%. Its sensitivity in diagnosing confirmed TB patients by molecular pathology reached 77.5%.

**Table 2:** Diagnostic performances of BNRS-dPCR assay for TB.

	**AUC (95% CI)**	**Criterion (copies/20 μL reaction mixture)**	**Sensitivity (%) (95% CI)**	**Specificity (%) ^c^ (95% CI)**	**LR+ (95% CI)**	**LR- (95% CI)**	**PPV (%) (95% CI)**	**NPV (%) (95% CI)**
**Total TB (*****n*** = **63)**								
IS*6110*-dPCR	0.84 (0.76–0.91)	> 4.2	60.3 (47.2–72.4)	97.7 (87.7–99.9)	25.9 (3.7–181.8)	0.4 (0.3–0.6)	97.4 (86.5–99.9)	62.7 (50.0–74.2)
IS*1081*-dPCR	0.84 (0.76–0.91)	> 1.7	57.1 (44.0–69.5)	100.0 (91.8–100.0)	–	0.4 (0.3–0.6)	100.0 (90.3–100.0)	61.4 (49.0–72.8)
IS*6110* and IS*1081-*dPCR^a^	0.75 (0.66–0.85)	> 4.2 and > 1.7	50.8 (37.9–63.6)	100.0 (91.8–100.0)	–	0.5 (0.4–0.6)	100.0 (89.1–100.0)	58.1 (46.1–69.5)
IS*6110* OR IS*1081-*dPCR^b^	0.82 (0.74–0.90)	> 4.2 or > 1.7	66.7 (53.7–78.0)	97.7 (87.7–99.9)	28.7 (4.1–200.5)	0.3 (0.2–0.5)	97.7 (87.7–99.9)	66.7 (53.7–78.0)
**Confirmed TB (*****n** =* **48)**								
IS*6110*-dPCR	0.86 (0.78–0.93)	> 4.2	62.5 (47.4–76.0)	97.7 (87.7–99.9)	26.9 (3.8–188.8)	0.4 (0.3–0.6)	96.8 (83.3 – 99.9)	70.0 (56.8–81.2)
IS*1081*-dPCR	0.84 (0.75–0.92)	> 1.7	58.3 (43.2–72.4)	100.0 (91.8–100.0)	–	0.4 (0.3–0.6)	100.0 (87.7–100.0)	68.3 (55.3–79.4)
IS*6110* and IS*1081-*dPCR	0.76 (0.66–0.86)	> 4.2 and > 1.7	52.1 (37.2–66.7)	100.0 (91.8–100.0)	–	0.5 (0.4–0.6)	100.0 (86.3–100.0)	65.2 (52.4–76.5)
IS*6110* OR IS*1081-*dPCR	0.83 (0.75–0.92)	> 4.2 or > 1.7	68.8 (53.7–81.3)	97.7 (87.7–99.9)	29.6 (4.2–207.0)	0.3 (0.2–0.5)	97.1 (84.7–99.9)	73.7 (60.3–84.5)
**Clinically suspected TB (*****n** =* **15)**								
IS*6110*-dPCR	0.77 (0.60–0.95)	> 4.2	53.3 (26.6–78.7)	97.7 (87.7–99.9)	22.9 (3.1–168.5)	0.5 (0.3–0.8)	88.9 (51.8–99.7)	85.7 (72.8–94.1)
IS*1081*-dPCR	0.85 (0.72–0.98)	> 1.7	53.3 (26.6–78.7)	100.0 (91.8–100.0)	–	0.5 (0.3–0.8)	100.0 (63.1–100.0)	86.0 (73.3–94.2)
IS*6110* and IS*1081-*dPCR	0.73 (0.56–0.91)	> 4.2 and > 1.7	46.7 (21.3–73.4)	100.0 (91.8–100.0)	–	0.5 (0.3–0.9)	100.0 (59.0–100.0)	84.3 (71.4–93.0)
IS*6110* OR IS*1081-*dPCR	0.79 (0.63–0.95)	> 4.2 or > 1.7	60.0 (32.3–83.7)	97.7 (87.7–99.9)	25.8 (3.6–187.0)	0.4 (0.2–0.8)	90.0 (55.5–99.7)	87.5 (74.8–95.3)
**Confirmed TB by molecular pathology (*****n** =* **40)**								
IS*6110*-dPCR	0.90 (0.84–0.97)	> 4.2	72.5 (56.1–85.4)	97.7 (87.7–99.9)	31.2 (4.5–218.3)	0.3 (0.2–0.5)	96.7 (82.8–99.9)	79.2 (65.9–89.2)
IS*1081*-dPCR	0.89 (0.81–0.96)	> 1.7	67.5 (50.9–81.4)	100.0 (91.8–100.0)	–	0.3 (0.2–0.5)	100.0 (87.2–100.0)	76.8 (63.6–87.0)
IS*6110* and IS*1081-*dPCR	0.81 (0.71–0.89)	> 4.2 and > 1.7	62.5 (45.8–77.3)	100.0 (91.8–100.0)	–	0.4 (0.3–0.6)	100.0 (85.8–100.0)	74.1 (61.0–84.7)
IS*6110* OR IS*1081-*dPCR	0.88 (0.79–0.94)	> 4.2 or > 1.7	77.5 (61.5–89.2)	97.7 (87.7–99.9)	33.2 (4.8–232.9)	0.2 (0.1–0.4)	96.9 (83.8–99.9)	82.4 (69.1–91.6)

^a^IS*6110* and IS*1081*-dPCR is positive if both IS*6110* and IS*1081* > their cut-off values, and negative if either IS*6110* or IS*1081* ≤ their cut-off values. ^b^IS*6110* OR IS*1081*-dPCR is positive if either IS*6110* or IS*1081* > their cut-off values, and negative if both IS*6110* and IS*1081* ≤ their cut-off values. ^c^Specificity was calculated in the non-TB group (N = 43). AUC, area under curve; CI, confidence interval; LR+, positive likelihood ratio; LR-, negative likelihood ratio; PPV, positive predictive value; NPV, negative predictive value.

### 3.4 Sensitivity comparison of BNRS-dPCR assay with routine diagnostic tests

The positive detection rates of different tests in TB patients are listed in Table 3. BNRS IS*6110* OR IS*1081*-dPCR showed comparable sensitivity to *M.tb* molecular detection using FFPE biopsy tissue (69.6% vs.71.4%, *P* = 1.000), and significantly higher sensitivity than AFB test using FFPE biopsy tissue (69.6% vs. 39.3%, *P* < 0.001). It exhibited higher sensitivity compared to routine etiological tests, including smear microscopy (58.8% vs. 5.9%), mycobacterial culture (52.9% vs. 17.6%), and Xpert (67.9% vs. 21.4%) using sputum samples, as well as smear microscopy (66.7% vs. 8.3%), mycobacterial culture (66.7% vs. 25.0%), and Xpert (72.2% vs. 22.2%) using BALF samples. When compared with immunological tests, the sensitivity of BNRS IS*6110* OR IS*1081*-dPCR was slightly lower than that of peripheral blood IGRA (66.7% vs. 81.3%, *P* = 0.143), and slightly higher than that of TB antibody detection in peripheral blood (60.0% vs. 40.0%, *P* = 0.289).

**Table 3:** Sensitivity comparisons of BNRS-dPCR assay with conventional tests in diagnosis of TB.

**Tests**	**Number of TB patients**	**Sensitivity % (n/N)**			***P*-value ^b^**
**BNRS IS*****6110*** **OR IS*****1081*****-dPCR**^a^ **compared with biopsy pathology**					
BNRS IS*6110* OR IS*1081*-dPCR vs. *M.tb* molecular detection using FFPE biopsy tissue	56	69.6% (39/56)	vs.	71.4% (40/56)	1.000
BNRS IS*6110* OR IS*1081*-dPCR vs. AFB test using FFPE biopsy tissue	56	69.6% (39/56)	vs.	39.3% (22/56)	< 0.001
**BNRS IS*****6110*** **OR IS*****1081*****-dPCR compared with routine etiological tests**					
BNRS IS*6110* OR IS*1081*-dPCR vs. sputum smear microscopy	17	58.8% (10/17)	vs.	5.9% (1/17)	0.004
BNRS IS*6110* OR IS*1081*-dPCR vs. sputum mycobacterial culture	17	52.9% (9/17)	vs.	17.6% (3/17)	0.109
BNRS IS*6110* OR IS*1081*-dPCR vs. sputum Xpert MTB/RIF	28	67.9% (19/28)	vs.	21.4% (6/28)	0.002
BNRS IS*6110* OR IS*1081*-dPCR vs. BALF smear microscopy	12	66.7% (8/12)	vs.	8.3% (1/12)	0.039
BNRS IS*6110* OR IS*1081*-dPCR vs. BALF culture	12	66.7% (8/12)	vs.	25.0% (3/12)	0.063
BNRS IS*6110* OR IS*1081*-dPCR vs. BALF Xpert MTB/RIF	18	72.2% (13/18)	vs.	22.2% (4/18)	0.035
**BNRS IS*****6110*** **OR IS*****1081*****-dPCR compared with immunological tests**					
BNRS IS*6110* OR IS*1081*-dPCR vs. peripheral blood IGRA	48	66.7% (32/48)	vs.	81.3% (39/48)	0.143
BNRS IS*6110* OR IS*1081*-dPCR vs. TB antibody in peripheral blood	20	60.0% (12/20)	vs.	40.0% (8/20)	0.289

^a^The cut-off values of BNRS IS*6110* OR IS*1081*-dPCR assay were 4.2 and 1.7 copies/20μL reaction mixture, respectively. One of them was positive meant the dPCR result was positive. ^b^McNemar test was used to determine the significance between BNRS IS*6110* OR IS*1081*-dPCR assay and other tests. FFPE, formalin-fixed paraffin-embedded; AFB, acid-fast bacilli; BALF, bronchoalveolar lavage fluid; IGRA, interferon-gamma release assays; n/N, the number of samples tested positive/the total number of samples.

### 3.5 Incremental diagnostic value of BNRS-dPCR in TB patients underwent biopsy

The positive proportion of BNRS IS*6110* OR IS*1081*-dPCR in TB cases with negative AFB test results and negative *M.tb* molecular detection results using FFPE biopsy tissue was 55.9% (19/34) and 50.0% (8/16), respectively. Combination of conventional biopsy pathology and BNRS-dPCR increased the positive detection rate in TB patients, with 39.3% for biopsy AFB test alone and 73.2% for it plus BNRS-dPCR, and 71.4% for biopsy *M.tb* molecular detection alone and 85.7% for it plus BNRS-dPCR (Table 4).

**Table 4:** BNRS-dPCR combined with conventional biopsy pathology increased the detection rate of TB.

**Tests**	**Specimens examined**	**Positive cases**	**Increased positive cases**	**Positive rate**
**BNRS IS*****6110*** **OR IS*****1081*****-dPCR assay**^a^ **in TB patients with negative biopsy results for** ***M.tb***				
BNRS IS*6110* OR IS*1081*-dPCR assay in TB patients with negative AFB test results using FFPE biopsy tissue	34	19	-	55.9%
BNRS IS*6110* OR IS*1081*-dPCR assay in TB patients with negative *M.tb* molecular detection results using FFPE biopsy tissue	16	8	-	50.0%
**Combination of BNRS IS*****6110*** **OR IS*****1081*****-dPCR assay with conventional biopsy pathology in TB patients**				
AFB test using FFPE biopsy tissue	56	22	-	39.3%
AFB test using FFPE biopsy tissue + BNRS IS*6110* OR IS*1081*-dPCR assay	56	41	19	73.2%
*M.tb* molecular detection using FFPE biopsy tissue	56	40	-	71.4%
*M.tb* molecular detection using FFPE biopsy tissue + BNRS IS*6110* OR IS*1081*-dPCR assay	56	48	8	85.7%

^a^The cut-off values of BNRS IS*6110* OR IS*1081*-dPCR assay were 4.2 and 1.7 copies/20 μL reaction mixture, respectively. One of them was positive meant the dPCR result was positive. AFB, acid-fast bacilli; FFPE, formalin-fixed paraffin-embedded.

## 4 Discussion

As shown in the results, BNRS IS*6110* OR IS*1081*-dPCR demonstrated considerable sensitivity in comparison with conventional diagnostic tests. We also assessed the agreement between BNRS IS*6110* OR IS*1081*-dPCR and these tests. The results indicated that BNRS IS*6110* OR IS*1081*-dPCR exhibited a higher agreement with biopsy pathology, with a *Kappa* value of 0.270 and 0.308 for agreement with molecular detection of *M.tb* and AFB detection using FFPE biopsy tissue, respectively (*P* = 0.043 and 0.005, respectively) (Supplementary Table S1). This can be attributed to the fact that BNRS is most similar to biopsy samples in terms of location and type compared to sputum, blood and BALF samples.

Considering that approximately a quarter of the global population is estimated to be affected by latent tuberculosis infection (LTBI), the detection results of BNRS IS*6110* OR IS*1081*-dPCR in the LTBI population are also a significant concern. IGRA is one of the effective methods recommended by WHO guidelines for LTBI detection, but it cannot distinguish between LTBI and active TB. Our findings revealed that the positive rate of BNRS IS*6110* OR IS*1081*-dPCR assay was significantly higher in non-TB patients with positive IGRA results (37.9%, 11/29) compared to those with negative IGRA results (2.3%, 1/43) (*P* < 0.001). This result suggests that the small amount of *M.tb* nucleic acid in the lung tissue of LTBI cases can be detected by the ultra-sensitive digital PCR method. Additionally, we observed that the number of target copies detected in 63 TB patients was significantly higher than that in 29 non-TB patients with positive IGRA results: median (25% percentile, 75% percentile), IS*6110*, 7.8 (2.2, 58.0) vs. 3.0 (0.9, 8.1) copies/20 μL reaction mixture, *P* = 0.005; IS*1081*, 2.7 (1.0, 19.1) vs. 0.9 (0.4, 2.2) copies/20 μL reaction mixture, *P* = 0.001. This finding is consistent with previous research suggesting that there is a pathogenetic continuum from *M.tb* exposure to infection to TB disease, individuals in different states have different bacterial loads, the trend of infection outcome depends on changes in the host immunity, and TB transmission can occur during the subclinical period (Pai and Behr, 2016; Wang et al., 2023). Subsequent in-depth research with larger sample size is needed to ascertain whether the copy number of target genes detected by dPCR can differentiate between LTBI and TB, appropriate threshold, and specific discriminative performance. Hence, patients with TB or other diseases combined with TB or LTBI may yield positive results in BNRS IS*6110* OR IS*1081*-dPCR testing. Healthcare professionals should make comprehensive judgments by considering other test results such as histopathology to avoid overlooking other serious conditions like lung cancer.

The results of BNRS IS*6110* OR IS*1081*-dPCR in non-TB patients with a history of TB are also noteworthy. In this study, ten non-TB patients with a known TB history were excluded due to positive IGRA results (1 case had completed at least 1 year of anti-TB treatment, while the other 9 cases had completed anti-TB treatment for decades). After analyzing the BNRS IS*6110* OR IS*1081*-dPCR results of these patients, we found that the positive rate was 50% (5/10) with low target level in most cases. Another study also reported that a small percentage (4%, 3/82) of non-TB participants with a known TB history yielded Xpert MTB/RIF Ultra trace outcomes from sputum samples (Wang et al., 2022). Furthermore, it was reported that non-TB patients with a previous TB history exhibited a significantly higher positive rate of IGRA compared to those without (84.3% vs. 26.9%, *P* < 0.001) (Kim et al., 2011). It remains unclear whether these non-TB patients had newly acquired *M.tb* infection or if there were still replicating or dormant *M.tb* bacteria and its nucleic acid fragments in the lung tissue due to incomplete anti-TB treatment in the past.

In this study, although BNRS IS*6110* OR IS*1081*-dPCR showed comparable sensitivity to molecular methods in FFPE biopsy samples (69.6% vs. 71.4%, *P* = 1.000), BNRS-dPCR yielded positive results in some patients with negative biopsy pathological results but diagnosed as TB through other tests. Specifically, among TB cases with negative molecular detection of *M.tb* results and negative AFB test results using FFPE biopsy tissue, the positive proportion of BNRS-dPCR was 50.0% and 55.9% respectively, suggesting that combination of conventional biopsy pathology and BNRS IS*6110* OR IS*1081*-dPCR has the potential to increase the etiological diagnosis rate in TB patients. Indeed, in this study, the rate increased from 39.3% for biopsy AFB test alone to 73.2% when combined with BNRS-dPCR, and from 71.4% for biopsy *M.tb* molecular detection alone to 85.7% when combined with BNRS-dPCR, respectively. This ability can be partly attributed to the utilization of robust physical lysis methods for *M.tb* DNA extraction along with the application of ultra-sensitive digital PCR technology. But more importantly, as previously mentioned, the outer wall of the puncture needle had a larger surface area compared to the needle groove containing the biopsy tissue. This difference enabled the cellular residues adhered to the outer wall of the needle to offer supplementary diagnostic value.

In conclusion, our study preliminarily revealed that the CT-guided percutaneous BNRS IS*6110* OR IS*1081*-dPCR test is an effective auxiliary diagnostic approach for TB. The utilization of this method can convert previously discarded materials into valuable resources, augment the diagnostic yield of biopsy procedures, and improve the diagnosis of TB. This holds great significance for patients whose diagnosis is still ambiguous despite undergoing multiple examinations including biopsy. However, due to the limited sample size in this study, further research with larger sample size and encompassing different infection stages is needed to better elucidate its potential application value in diagnosis and pathogenesis of TB.

## Data availability statement

The raw data supporting the conclusions of this article will be made available by the authors, without undue reservation.

## Ethics statement

The studies involving humans were approved by Ethics Committee of Beijing Chest Hospital, Capital Medical University. The studies were conducted in accordance with the local legislation and institutional requirements. The participants provided their written informed consent to participate in this study.

## Author contributions

ZL: Data curation, Formal analysis, Funding acquisition, Investigation, Methodology, Project administration, Writing—original draft, Conceptualization, Writing—review & editing. BW: Data curation, Investigation, Resources, Writing—original draft, Writing—review & editing, Methodology. BD: Investigation, Writing—review & editing. QS: Investigation, Writing—review & editing. DW: Resources, Writing—review & editing. RW: Investigation, Writing—review & editing. CL: Resources, Writing—review & editing. CZ: Funding acquisition, Investigation, Writing—review & editing. HJ: Investigation, Writing—review & editing. AX: Investigation, Writing—review & editing. ZZ: Supervision, Writing—review & editing. LP: Conceptualization, Funding acquisition, Methodology, Supervision, Writing—review & editing. DH: Conceptualization, Resources, Supervision, Writing—review & editing, Methodology.
