# Supplementary material for: The incremental value of Mycobacterium tuberculosis trace nucleic acid detection in CT-guided percutaneous biopsy needle rinse solutions for the diagnosis of tuberculosis
Source: Front Microbiol. 2024 Feb 8;15:1335526. doi: 10.3389/fmicb.2024.1335526 (PMC10882560; doi:10.3389/fmicb.2024.1335526)
Supplement: Supplementary file 1 [file Table_1.DOCX]

Supplementary Material

# Supplementary Tables

**Table S1** Agreement between BNRS-dPCR assay and other tests in diagnosis of TB.

| Tests | Number of TB patients | Agreement | *Κappa*-value | *P*-value |
| --- | --- | --- | --- | --- |
| BNRS IS*6110* OR IS*1081*-dPCR ^a^ *vs.* *M.tb* molecular detection using FFPE biopsy tissue | 56 | 69.6% (39/56) | 0.270 | **0.043** |
| BNRS IS*6110* OR IS*1081*-dPCR *vs.* AFB test using FFPE biopsy tissue | 56 | 62.5% (35/56) | 0.308 | **0.005** |
| BNRS IS*6110* OR IS*1081*-dPCR *vs.* sputum smear microscopy | 17 | 47.1% (8/17) | 0.084 | 0.388 |
| BNRS IS*6110* OR IS*1081*-dPCR *vs.* sputum mycobacterial culture | 17 | 41.2% (7/17) | -0.133 | 0.453 |
| BNRS IS*6110* OR IS*1081*-dPCR *vs.* sputum Xpert MTB/RIF | 28 | 39.3% (11/28) | -0.008 | 0.944 |
| BNRS IS*6110* OR IS*1081*-dPCR *vs.* BALF smear microscopy | 12 | 25.0% (3/12) | -0.174 | 0.140 |
| BNRS IS*6110* OR IS*1081*-dPCR *vs.* BALF culture | 12 | 58.3% (7/12) | 0.286 | 0.157 |
| BNRS IS*6110* OR IS*1081*-dPCR *vs.* BALF Xpert MTB/RIF | 18 | 16.7% (3/18) | -0.337 | 0.017 |
| BNRS IS*6110* OR IS*1081*-dPCR *vs.* peripheral blood IGRA | 48 | 64.6% (31/48) | 0.105 | 0.433 |
| BNRS IS*6110* OR IS*1081*-dPCR *vs.* TB antibody in peripheral blood | 20 | 60.0% (12/20) | 0.231 | 0.264 |

^a^ The cut-off values of BNRS IS*6110* OR IS*1081*-dPCR assay were 4.2 and 1.7 copies/20µL reaction mixture, respectively. One of them was positive meant the dPCR result was positive.

FFPE, formalin-fixed paraffin-embedded; AFB, acid-fast bacilli; BALF, bronchoalveolar lavage fluid; IGRA, interferon-gamma release assays.
